# Effects of magnetized water on ovary, pre-implantation stage endometrial and fallopian tube epithelial cells in mice

**Published:** 2014-04

**Authors:** Leili Hafizi, Mostafa Gholizadeh, Mohammad Karimi, Golkoo Hosseini, Hesam Mostafavi-Toroghi, Mehdi Haddadi, Amin Rezaiean, Mahmoud Ebrahimi, Neda Emami Meibodi

**Affiliations:** 1 *Department of Obstetrics and Gynecology, Mashhad University of Medical Sciences, Mashhad, Iran.*; 2 *Department of Chemistry, Faculty of Basic Sciences, Ferdowsi University, Mashhad, Iran.*; 3 *Faculty of Medicine, Mashhad University of Medical Sciences, Mashhad, Iran.*; 4 *Biochemistry of Nutrition Research Center, Faculty of Medicine, Mashhad University of Medical Sciences, Mashhad, Iran.*; 5 *Department of Microbiology, Faculty of Medicine, Zahedan University of Medical Sciences, Zahedan, Iran.*; 6 *Cardiovascular Research Center, Mashhad University of Medical Sciences, Mashhad, Iran.*; 7 *Department of Biomedical Engineering, Engineering Faculty, Islamic Azad University, Mashhad, Iran.*

**Keywords:** *Water*, *Fertility*, *Mice*, *Fallopian tubes*, *Endometrium*, *Epithelial cells*

## Abstract

**Background:** Magnetized water has made many improvements in industry, agriculture and medicine. However its utilization in medicine still remains controversial.

**Objective:** In this study we aimed to investigate the effects of magnetized water on height of epithelial cells in pre-implantation stage endometrium and fallopian tube and number of corpus lutea in female mice.

**Materials and Methods: **Eighty female NRMI mice were recruited to this experimental study and randomly divided into two groups: the control group which drank normal water and the experimental (case) group which drank magnetized water for 2 weeks. Super-ovulation was induced in these mice and then they were mated with male mice as well. Samples of ovary, uterus and fallopian tube were obtained at the pre-implantation stage. Then, after preparation, the number of corpus lutea in each ovary was counted and the height of fallopian and endometrial epithelial cells was measured by light microscopy.

**Results: **Data analysis showed a significant increase in the mean number of corpus lutea and the height of epithelial cells in fallopian tube comparing the case with the control group (p=0.01, p=0.002 respectively) whereas uterus epithelial cells of the case group showed insignificant increase in height, in compare with the control group (p=0.052).

**Conclusion:** Our results suggest that magnetized water intake increases the number of corpus lutea and the height of fallopian tube epithelial cells. Further research is needed to determine whether this will increase in the success rate of fertility.

## Introduction

Infertility is one of the high yield medical problems (1). Assisted reproductive techniques (ART) are well known therapeutic options from which in vitro fertilization (IVF) involves extracting egg from the woman’s ovaries, egg and sperm zygosis, and transferring the fertilized egg to the mother’s womb. Regarding the physical, mental and financial challenges of IVF failure, finding the methods that can improve the success rate of IVF is necessary (2,3). Passing water through a magnetic field can change its physical, chemical and bacteriological characteristics in a way that converts it to a beneficial substance for the industry, agriculture and medicine. 

By flowing water through a permanent magnetic field, since they are perpendicular to magnetic field lines (θ=90), fluid orients accordingly. This causes the positive and negative particles to separate and rearrange to form a new structure. The final result is called “Magnetized Water” (4). Several studies have demonstrated a lot of microscopic and macroscopic differences between magnetized water and normal water; which include the surface tension, contact angel, viscosity, electrical conductivity, pH, etc. (5).

Surface tension in magnetized water is reduced by 10-12% whilst its velocity is increased in compare with regular water. Therefore it’s penetration into cell wall would be facilitated which can accelerate ordinary diffusion of water that is vital for growth of different organs (6). Nakagawa *et al* showed that the dissolution of oxygen into water is significantly accelerated in the presence of a magnetic field (7). Numerous animal studies have proved that electromagnetic fields have potential effects on cellular mechanism, development and growth especially in the reproductive system (8, 9). 

In some other studies the potential effect of magnetized water on biological cells have been demonstrated (10,11). One of the proliferative responses of the uterine epithelium is hyperplasia and stratification of the epithelial cells which affect the implantation phase. Therefore increasing the height of endometrial and fallopian epithelial cells can improve the proliferative indices (12,13). Previously it has been demonstrated that exposure to extremely low-frequency electromagnetic field (ELF-EMF) can increase height of fallopian tube epithelial cells. 

Although the exact mechanism of increasing epithelial cell height is not clear yet, it has been hypothesized that it could be due to increased permeability of cellular membrane to small molecules, especially Ca^2+^ current, free radical chemical reactions and increase in superoxide dismutase activity in the liver (14). Previous studies have investigated the effect of ELF-EMF on male and female fertility indices while, in the present study, we aimed to investigate the effects of magnetized water on the height of endometrial epithelial cells, fallopian tube epithelium and the number of corpus lutea before implantation in female mice.

## Materials and methods

This experimental study was conducted under approval of Ethics Committee of Mashhad University of Medical Sciences. In this study a total of 80 female Naval Medical Research Institute (NMRI) mice were obtained from the Razi Institute, Iran and housed under standard laboratory conditions. Subjects were kept at constant room temperature (21±2^o^C) under a normal 12-hours light/ 12-hours dark cycle with free access to food. All animal experiments carried out in accordance with the European Communities Council Directive of 24 November 1986 (86/609/EEC) to minimize damage to the animals. 

Mice were randomly divided into two groups: control group drinking normal water and the case group drinking magnetized water for 2 weeks. Water magnetizing process was done based on a standard method that has been used previously (14,15). The devices contain two compartments with the total capacity of 4 liters, an alternator to circulate the water and the part that creates magnetic field for magnetizing water. Three-time distilled water was obtained from “Toos Power Plant” (Mashhad-Iran) and poured into device’s water compartment and was exposed to a magnetic field with the intensity of 4000 G at a temperature of 37.5^o^C for 4 hours. 

Then the water was magnetic and could preserve its condition for 72 hours. Mice of both groups were stimulated to have enhanced ovulation by injecting 10 IU of pregnant mare’s serum gonadotropin (PMSG) (from Folligon, Intervet, and Netherlands) and then 10 IU human chorionic gonadotropin (hCG) (from Oregone, Netherlands). Forty-eight hours later to injection of 10 IU hCG, female mice were contacted with male mice during the night. The morning of the next day pregnant mice of both groups have been selected regarding to vaginal plug and then separated. The number of pregnant mice in the case and control group was 20 and 25 respectively. 

102 hours after hCG injection, cervical dislocation was conducted on pregnant mice and their womb has been removed. Number of fetuses has been measured by flushing the uterus horn. Number of corpus lutea was counted in microscopic preparations of the ovary samples. The ratio of the fetuses to the corpus lutea count can be used as an index for the fertility. After taking samples from uterus and fallopian tubes and tissue preparing, the tissues were cut by the microtome (Shandon AS 325) with the thickness of 5μ. Five sections from each sample were stained using hematoxylin and eosin protocol. 

**Figure 1:**
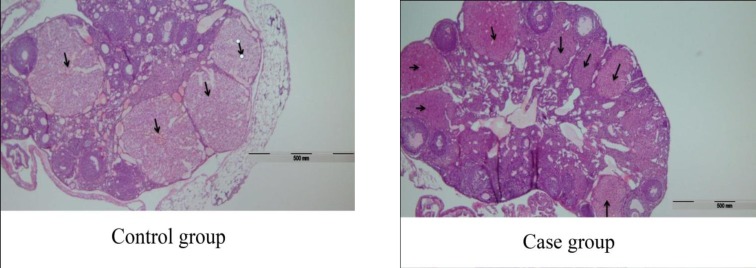
Microscopic view of corpus lutea in case vs. control group (x100). The number of corpus lutea in the case group had a significant increase in compare with the control group

Then the height of epithelial cells was measured using a light microscope (Nikon, Japan) equipped with cell measurement software, that is capable of measuring distance between marked base and apex in micrometers. Measurements were done in 100x magnification and in each slide, 100 cells were randomly measured. A comparison between control group and the case group was carried out (Figure 1).

**Statistical analysis**

Results are reported as mean±SD. Data analysis was done by Statistical Package for the Social Sciences software (SPSS, 18^th^ release). Between groups comparison was performed by using Student's *t* -test. A significance level of 0.05 (p<0.05) was considered.

## Results

Among the 40 mice in each case and control group, 20 and 25 mice became pregnant, respectively. The mean±SD number of corpus lutea in the case and the control group were 9±4 and 5±2, respectively. Data analysis showed that the number of corpus lutea in the case group had a significant increase in compare with the control group (p=0.01) (Figure 1). The mean±SD height of epithelial cells in fallopian tube of the case and the control group were 22.74±0.18 and 20.28±0.35 micrometers respectively. 

**Figure 2:**
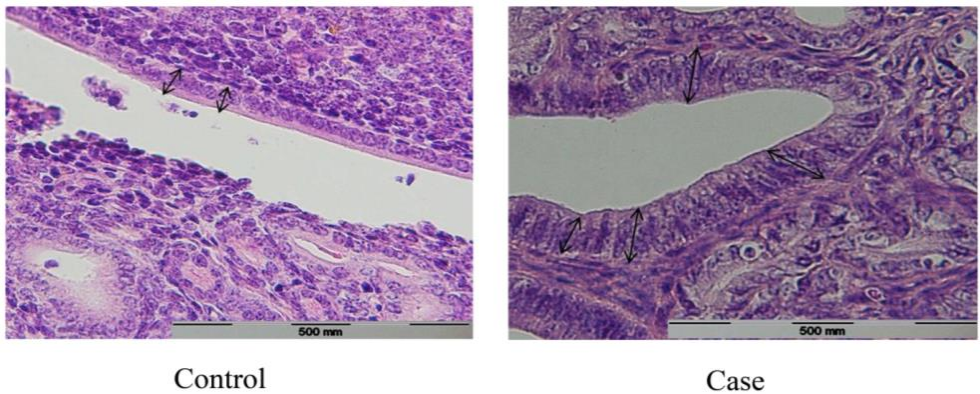
Microscopic view of fallopian tube epithelium in case vs. control group (x100). Height of epithelial cells in fallopian tube of the case group was significantly more than the control group

**Figure 3:**
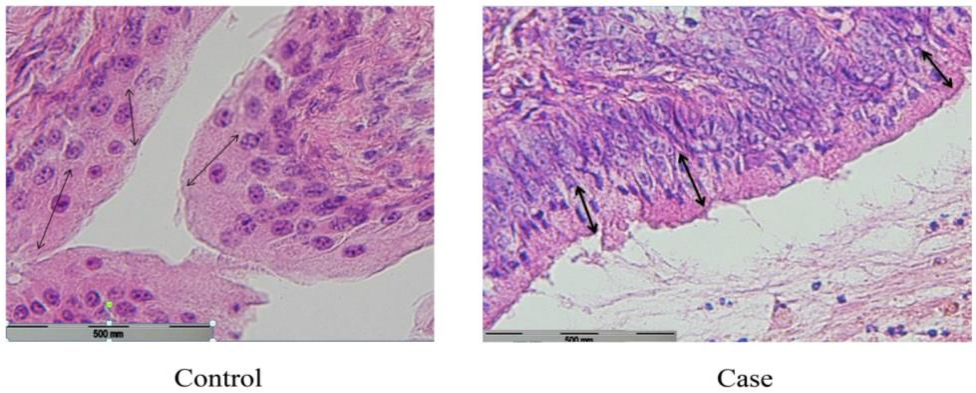
Microscopic view of endometrial epithelium in case vs. control group (x100). The mean±SD height of uterus epithelial cells of the case group was more than the control group

In this regard, the comparison of two groups demonstrated a significant increase in the case group (p=0.002) (Figure 2). The mean±SD height of uterus epithelial cells of the case and the control group were 20.48±0.43 and 19.17±0.11 micrometers respectively. Although the mean height of uterus epithelial cells in the case group has been increased, this was statistically insignificant (p=0.052) (Figure 3). The composition of nuclei in both groups was central.

## Discussion

In this study the effect of magnetized water on female reproductive system of mice was investigated. Our results showed a significant increase in the number of corpus lutea and the height of fallopian tube epithelium also an insignificant increase in the height of uterus endometrial epithelium in consequence of consuming magnetized water. During recent decades using magnetically treated water offered improvements in industry and agriculture such as scale reduction and increased crop yields (16,17). 

Despite numerous studies, application of magnetized water in medicine still remains controversial (18,19). Effectiveness of magnetized water in the prevention and treatment of atherosclerosis has been recently shown in some animal and human studies (-). However, its mechanism of action is not still well understood and various contradictory hypotheses have been proposed. It seems that this is the first study which investigates the probable effect of magnetized water on female mice reproductive system. Previously animal studies on female mice has showed that extremely low frequency electromagnetic field can increase the heights of epithelial cells in pre-implantation stage endometrium and fallopian tube (14, 24). 

Our results showed drinking magnetized water was effective in increasing the height of fallopian tube epithelium and the number of corpus lutea. The mechanisms of these effects are ambiguous. The potential hypotheses could be regarding to the effects of magnetized water on oxidative stress and cell growth which result in bio-activation improvement in epithelial cells.

**Magnetized water and oxidative stress**

Some animal studies have concluded that magnetized water could influence effectively on the oxidant-antioxidant balance, for instance it could decrease the amounts of Malondialdehyde (MDA), increase the superoxide dismutase activity in the heart, kidney and liver and also decrease the amounts of nitric oxide which all result in decreasing oxidative stress (10,11,25). Since the disturbing role of free radicals and oxidative stress in female reproduction has been proven, therefore, it could be assumed that this potential reduction of oxidative stress will result in improving the reproductive system (26, 27). Also, it has been demonstrated that intake of magnetized water will result in reduced DNA damage (28).

**Magnetized water and cell growth**

Magnetized water is probably effective in cell growth by increasing oxygen concentration, mineral solubility and accelerating transfer of water and nutrients in all compartments of body via improvement of permeability of cell wall as a consequence of decreased surface tension and electric conductivity (6, 7). The effect of magnetic field on intracellular fluid and substances results in enzyme activation and increased biochemical reactions in the cell (28). The reduction of suspended solids and pathogenic bacteria is another assumable factor that can explain this result. Gholizadeh *et al* has demonstrated that magnetized water can improve the growth and quality of the poultry (15).

Ma and colleagues have shown that magnetized water increased glutamate decarboxylase activity by 30%, which may have a role in mechanism of physiological and biological response of body to magnetized water (29). Eventually, although the mechanism is unclear but obviously magnetized water can influence the height of epithelial cells of fallopian tube. Regarding the role of fallopian tube epithelium in secreting colony stimulating factor 1 (CSF1), epidermal growth factor and interleukin 2, the effect of magnetized water on epithelial cells of the fallopian tube could be in favor of fertility (30, 31). 

On the other hand the uterine epithelial cells and corpus luteum play an essential role in the implantation phase. Increased number of corpus lutea in association with increased height of uterus epithelial cells may support the establishment of pregnancy (32). More studies regarding the effect of magnetized water on male reproductive system is suggested. Also a meticulous study regarding the effect of magnetized water on reactive oxygen species (ROS) concentration and sex-hormones profile is suggested.

**Limitations**

The hormonal profile which is a very important fertility index has not been assessed during this process. Also it would be a helpful parameter for finding the principles of this phenomenon. On the other hand potential side-effects of magnetized water should be studied.

## Conclusion

Magnetized water is an inexpensive, environment-friendly substance. Despite its ubiquity, there is relatively little scientific literature on magnetic water treatment. Our results demonstrated that intake of magnetized water affect the reproductive organs of pregnant female mice by increasing the number of yellow bodies and the height of fallopian tube epithelium in pre-implantation stage. It requires further investigations to determine whether the observed effect of magnetized water could increase fertility.
